# The hereditary spastic paraplegia protein strumpellin: Characterisation in neurons and of the effect of disease mutations on WASH complex assembly and function

**DOI:** 10.1016/j.bbadis.2012.10.011

**Published:** 2013-01

**Authors:** Caroline Freeman, Matthew N.J. Seaman, Evan Reid

**Affiliations:** a Department of Medical Genetics and Cambridge Institute for Medical Research, University of Cambridge, UK; b Department of Clinical Biochemistry and Cambridge Institute for Medical Research, University of Cambridge, UK

**Keywords:** WASH complex, Retromer, Strumpellin, Hereditary spastic paraplegia, Axon degeneration, Sorting nexin

## Abstract

Mutations in the gene encoding strumpellin cause autosomal dominant hereditary spastic paraplegia (HSP), in which there is degeneration of corticospinal tract axons. Strumpellin is a component of the WASH complex, an actin-regulating complex that is recruited to endosomes by interactions with the retromer complex. The WASH complex and its relationship to retromer have not been fully characterised in neurons, and the molecular pathological mechanism of strumpellin mutation is unclear. Here we demonstrate that the WASH complex assembles in the brain, where it interacts with retromer. Members of both complexes co-localise with each other and with endosomes in primary cortical neurons, and are present in somato-dendritic and axonal compartments. We show that strumpellin is not required for normal transferrin receptor traffic, but is required for the correct subcellular distribution of the β-2-adrenergic receptor. However, strumpellin disease mutations do not affect its incorporation into the WASH complex or its subcellular localisation, nor do they have a dominant effect on functions of the WASH complex, including regulation of endosomal tubulation, transferrin receptor traffic or β-2-adrenergic receptor localisation. Models of the WASH complex indicate that it contains a single strumpellin molecule, so in patients with strumpellin mutations, complexes containing wild-type and mutant strumpellin should be present in equal numbers. In most cell types this would provide sufficient functional WASH to allow normal cellular physiology. However, owing to the demands on membrane traffic imposed by their exceptionally long axons, we suggest that corticospinal neurons are especially vulnerable to reductions in functional WASH.

## Highlights

► We studied the spastic paraplegia protein strumpellin, a component of WASH complex. ► The WASH complex assembles in neurons where it interacts with retromer on endosomes. ► WASH and retromer are in axons, the site of hereditary spastic paraplegia pathology. ► Strumpellin regulates the subcellular distribution of the β-2-adrenergic receptor. ► Disease mutants do not have a dominant effect on cellular functions of strumpellin.

## 1 Introduction

Purposeful movement of the limbs requires rapid communication from brain to muscle. This is achieved in humans by a motor pathway comprising two classes of neurons, termed upper motor neurons (UMNs) and lower motor neurons (LMNs). The UMN cell bodies are in the brain motor cortex and the axons of these neurons form the corticospinal tracts, which project to the spinal cord. These axons synapse, directly or via interneurons, with LMNs in the spinal cord ventral horn. The LMN axons then run in peripheral nerves to innervate muscle.

The extreme length of the motor pathway axons (up to 1 m in humans) allows efficient signal transmission, but requires complex cellular systems to sort and transport cargoes to the axon terminus. Reflecting this vulnerability, degeneration of the corticospinal tract axons is a clinically significant feature in a number of neurological disorders, including subtypes of common neurodegenerative conditions such as multiple sclerosis and amyotrophic lateral sclerosis [1,2]. Corticospinal tract axonopathy is also the main pathological feature of a group of genetic disorders termed the hereditary spastic paraplegias (HSPs) [3,4]. As HSPs are single gene disorders, identification of the responsible genes has allowed the precise characterisation of proteins and biochemical pathways that are critical for corticospinal axon integrity.

More than 20 genes mutated in HSP have been identified [5]. Strikingly, the majority of the proteins they encode have a role in intracellular membrane traffic, with at least 8 HSP proteins localising to endosomes [6,7]. This endosomal group includes strumpellin, a 1159aa protein encoded by the SPG8 (KIAA0196) gene [8]. With the exception of a spectrin repeat domain, which is characteristically found in proteins that have a transient interaction with the cytoskeleton, strumpellin has no recognisable domains. However, the protein participates in the WASH complex, a recently described multimeric protein complex that acts at the interface between actin regulation and endosomal membrane dynamics [9,10].

Many classes of membrane receptor, including for example the transferrin receptor, are sorted away from early endosomes in tubular transport intermediates [11,12]. These tubules have been proposed to separate from the endosome as a result of the concerted action of an actin dependent pushing force generated by the WASH complex, a pulling force generated by the microtubule motor dynein, and membrane scission mediated by dynamin (which also interacts with the WASH complex) [9–11,13]. Thus cells lacking components of the WASH complex, including strumpellin, have increased endosomal tubulation, consistent with a defect in tubule fission from the endosome [9,10,13].

**Fig. 1:**
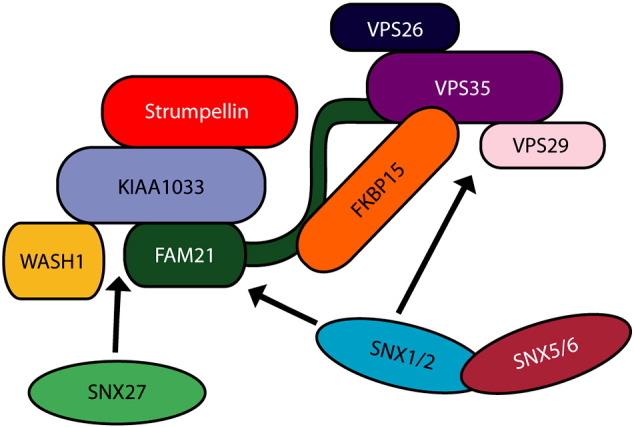
The WASH and retromer complexes. The WASH complex comprises the core proteins KIAA1033, strumpellin, Wash1 and FAM21. Wash1, FAM21 and strumpellin interact directly with KIAA1033, while the association between the WASH and retromer complexes occurs via the tail domain of FAM21. FKBP15, a protein of unknown function, interacts with both retromer and the FAM21 tail. Retromer consists of a core complex of VPS26, 29 and 35, with an associated sorting nexin dimer comprising a combination of SNXs 1, 2, 5 and 6. SNX27 has been reported as an additional interactor of the WASH complex. Interactions between the sorting nexins and the WASH and retromer complexes are indicated by arrows.

WASH consists of the core components Wash1 (Wiskott–Aldrich Syndrome Protein and SCAR Homologue 1), FAM21, KIAA1033 (also called SWIP; strumpellin and WASH-interacting protein), ccdc53 (coiled coil domain containing 53) and strumpellin, along with a number of accessory proteins (Fig. 1 and Table 1) [9,10,13–15]. Strumpellin participates in the complex via interactions with KIAA1033 (Fig. 1) [13]. In keeping with the role of the complex in regulating endosomal actin networks, Wash1 is a Nucleation Promoting Factor (NPF) that recruits and activates the actin-regulating Arp2/3 complex to create branched actin networks on endosomal subdomains [9,10]. FAM21 contains an actin capping protein binding motif, and has been proposed to control the activity of actin capping proteins, which regulate the stability and polymerisation capacity of actin fibres [16]. The precise role of strumpellin within the complex is not known.

The WASH complex is recruited to endosomes by the retromer complex [10,13]. Retromer was first characterised as mediating retrieval of certain receptors from endosomes to the trans-Golgi network. It is composed of two loosely-associated sub-complexes: a core complex of vacuolar sorting proteins (VPS) 35, 29 and 26, which is involved in cargo selection, and a dimeric sub-complex comprising a combination of sorting nexins 1 (SNX1), SNX2, SNX5 or SNX6, which are believed to provide a structural, membrane curvature-inducing activity [17,18]. Recruitment of the WASH complex to retromer is mediated by an interaction between VPS35 and the tail domain of FAM21 [15,19]. More recently, another sorting nexin, SNX27, has been identified as a binding partner of the WASH complex and as a putative cargo adaptor allowing the packaging of the beta-2-adrenergic receptor (β2AR) into retromer-positive endosomal tubules for recycling to the plasma membrane. This suggests that the WASH complex could be recruited by interactions with different adaptors to distinct endosomal tubules or membrane domains [20]. Despite the involvement of strumpellin and other members of the WASH and retromer complexes in neurodegenerative disease, many of these proteins have not been characterised in neuronal cells.

Three disease-causing mutations in strumpellin have been described, V626F, L619F and N471D, and all lie in highly conserved sequences [8]. In zebrafish, morpholino-mediated depletion of strumpellin caused a curly tail phenotype that was rescued by expression of wild-type but not disease-mutant human strumpellin, indicating that the mutants are non-functional. Expression of disease-mutant strumpellin on a wild-type background did not cause a phenotype, suggesting that the mutant proteins do not act by a dominant negative effect [8]. However, the relationship between the zebrafish phenotype and the molecular cell biological roles of strumpellin demonstrated in mammalian cells is not clear, and it is important to examine systematically whether strumpellin mutations affect, by a loss-of-function or dominant negative effect, the known functions of the protein in mammalian cells.

Here, we demonstrate that the WASH and retromer complexes interact in neuronal tissue, and that they localise to the endosomes in primary neurons. We further show co-localisation in primary neurons between pairs of WASH complex members, indicating that the role of the WASH complex in neurons is likely to be analogous to its function in other cell types. In addition, we show that strumpellin mutations do not affect the interactions of strumpellin within the WASH complex or the localisation of strumpellin in primary neurons or HeLa cells, nor do they have a dominant negative effect on endosomal tubulation or transferrin trafficking. These studies provide fundamental observations on strumpellin and the WASH complex in neuronal cells, and give further insights into the likely molecular pathological mechanism of strumpellin mutation.

## 2 Materials and methods

### 2.1 Antibodies and constructs

Antibodies against VPS26 and SNX1 used for both Western blotting and immunofluorescence, were as previously described [21]. The anti-Wash1 (FAM39B) polyclonal antibody was purchased from Sigma. The anti-strumpellin (C-14) antibody was from Santa Cruz Biotechnology. The anti-MAP2 monoclonal antibody was purchased from Abcam. Anti-EEA1 and anti-GM130 monoclonal antibodies were both from BD Biosciences. Monoclonal anti-myc (4A6) was from Millipore, and monoclonal anti-GFP (3E6) from Invitrogen. The rabbit anti-GFP antibody was described in [22]. Monoclonal anti-HA (16B12) was from Covance. Polyclonal antibodies against FAM21 and FKBP15 were from Santa Cruz and Abcam respectively. The strumpellin-myc construct was generated by subcloning the cDNA coding for strumpellin from the strumpellin-GFP construct described in Harbour et al., 2010 [13], into a modified pEGFP N3 vector where the GFP ORF had been replaced by DNA coding for 6 myc epitopes in tandem. The DNA for 6 myc epitopes was sourced from the Vps10-myc construct described in Seaman et al., 1997 [23] and was excised from the Vps10-myc construct by digestion with Dra1 and Not1 and then first cloned into pGEX cut with Sma1 and Not1. Following digestion of the pGEX-6myc construct with BamH1 and Not1 the fragment coding for the 6 myc epitopes was cloned into pEGFP N3 that had been digested with BamH1 and Not1 to excise the GFP ORF. GFP-tagged KIAA1033 and FAM21 constructs were as described previously [13]. HA-β2AR was purchased from the Missouri S&T cDNA Resource Center. The HA-SNX27 construct was a generous gift from Mark von Zastrow (Dept. of Psychiatry, UCSF).

### 2.2 Cell culture

Adherent HeLa cells were cultured at 37 °C in Dulbecco's modified Eagle's medium (Sigma) containing 10% foetal calf serum (FCS), penicillin/streptomycin and l-glutamine.

To generate primary cortical neurons rat embryos were obtained from pregnant Wistar rats on embryonic Day 18. Euthanasia was performed by CO_2_ asphyxiation and cervical dislocation. Embryos were decapitated, and the brains removed. Cortices were dissected from embryonic brains under a dissection microscope, and placed in Hanks Balanced Salt Solution (HBSS) media (Invitrogen) on ice.

Glass coverslips for primary neuronal culture were prepared by incubation at 37 °C overnight in a solution of 1 × poly-d-lysine (150 μg/ml) (Sigma), washed twice in sterile-filtered H_2_O and left to dry before use. For cell plating, rat embryonic cortices were washed in HBSS and then incubated in 5 ml trypsin media (HBSS + 0.25% trypsin + 0.1% DNAse) at 37 °C for 10 min. Trypsinised cortices were then washed three times in Wash medium (Neurobasal media (Invitrogen) + 10% FCS + 0.1% DNAse). Cortices were pelleted by centrifugation (1000 rpm, 5 min, 4 °C), and resuspended in neurobasal plating medium (Neurobasal media plus 1 × B27 serum-free supplement (Invitrogen), penicillin/streptomycin and l-glutamine). Cortical tissue was mechanically disrupted to achieve a single cell suspension by passing through fire-polished glass pipettes of decreasing bore size. This suspension was then passed through a 70 μm sieve strainer (BD Biosciences), and the cells pelleted by centrifugation (1500 rpm, 5 min, 4 °C). Cells were resuspended in plating medium, counted and plated onto glass coverslips at 1.25 × 105 per coverslip. Primary neurons were maintained at 37 °C/5% CO_2_ and re-fed every 3 days. For re-feeding, half the medium on each coverslip was removed and replaced with fresh plating medium.

### 2.3 Transfection

Rat primary cortical neurons were transfected using the Effectene Kit (Qiagen) following the manufacturer's protocol. Before transfection, 0.5 ml of neurobasal medium was removed from each coverslip and reserved. Transfection mixes were added to the remaining neurobasal medium in each well, and the neurons were incubated at 37 °C for 6–12 h. Post-transfection, 0.5 ml fresh neurobasal medium and 0.5 ml reserved neurobasal medium were added to each coverslip. Neurons were allowed to recover at 37 °C for 24 h before fixation.

HeLa cells were transfected using the HeLa Monster kit (Mirus Bio) following the manufacturer's protocol, and incubated with transfection reactions for 24 h. Where constructs were co-transfected, equal concentrations of each construct were used to make up the total recommended amount of DNA per transfection reaction.

For siRNA transfection, cells were transfected with the KIAA0196 (strumpellin; catalogue reference L-021222-01) or CLTC (clathrin heavy chain, catalogue reference L-004001-01) ON-TARGETplus siRNA smartpools (Dharmacon) at a concentration of 5 μM, using Oligofectamine transfection reagent (Invitrogen). Transfection of siRNA duplexes was carried out using a 5 day protocol, as previously described [24]. The efficiency of siRNA knock-down was verified by immunoblotting cell lysates with an antibody against the relevant protein. Where cells were additionally transfected with a DNA construct during the knockdown, the DNA transfection was carried out on Day 5, and harvesting on Day 6.

### 2.4 Co-immunoprecipitation experiments

HeLa cells used in co-IPs were harvested in PBS lysis buffer + 2% Triton TX-100 (plus protease inhibitors). Where transfected proteins were analysed, the cells were harvested 24 h after transfection. The IP protocol was as previously described [25]. For IP, anti-VPS26 was used at a concentration of 1:500; anti-Wash1 at 1:300; anti-strumpellin at 1:100; anti-SNX1 at 1:400; and anti-GFP at 1:1000.

### 2.5 Immunofluorescence microscopy

HeLa cells and neuronal cells were prepared for immunofluorescence by fixation with 3.8% formaldehyde in PBS and permeabilisation with 0.1% Triton TX-100 in PBS. Subsequent procedures were carried out as described previously [24,25]. Cells were analysed using a Zeiss LSM510 confocal microscope or using a Zeiss Axioplan light microscope and Simple PCI Software. Images were processed using the Adobe Photoshop and Illustrator programs. For SNX1 tubule counting and scoring, 3 experiments were carried out in which 30 cells were scored per experimental condition, and microscopy and tubule scoring were carried out as a double-blind procedure. Cells were imaged on a Zeiss Axioplan light microscope.

### 2.6 Transferrin uptake and recycling assays

Transferrin uptake and recycling assays were performed as in [26]. Briefly, adherent HeLa cells transfected with either siRNA or DNA were trypsinised, resuspended in Optimem and incubated on ice with Alexa-Fluor transferrin-647 (Invitrogen). For the uptake assay, cell aliquots were then incubated at 37 °C for 0, 5, 10 or 20 min before fixation. For the recycling assay, incubation of cells for 30 min at 37 °C to internalise transferrin was followed by the addition of unlabelled transferrin to stimulate recycling of fluorescent transferrin to the cell surface, and cells were then incubated and fixed at time points up to 20 min. Cells were fixed in a solution of 3.8% formaldehyde in PBS and analysed using a BD Facscalibur FACS machine. Depletion of the clathrin heavy chain was used as a positive control in these assays.

## 3 Results

### 3.1 The WASH complex assembles in the brain and interacts with retromer

**Fig. 2:**
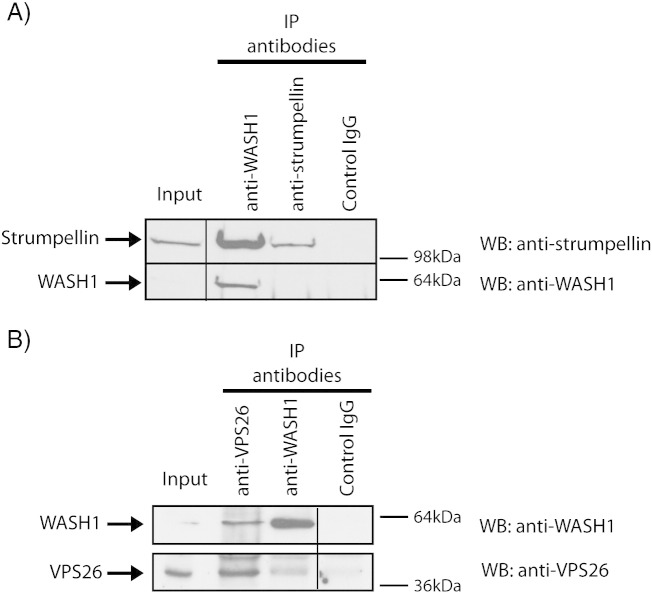
WASH complex is present in the brain and interacts with retromer. (A) and (B). Rat brain lysates were immunoprecipitated (IP) with the antibodies indicated, then immunoblotted (WB) with the antibodies shown. Spurious lanes on the blots have been spliced out, as indicated by the vertical lines. The strumpellin antibody appears relatively inefficient for IP, but sensitive for WB, whereas the WASH1 antibody is efficient for IP but relatively insensitive for WB.

To examine whether the WASH complex assembles in tissue relevant to the HSP phenotype, we began by confirming that Wash1 and strumpellin participate in a complex in the brain. We immunoprecipitated endogenous Wash1 from rat brain lysate, and found that endogenous strumpellin was co-immunoprecipitated (Fig. 2A). As the interaction between Wash1 and strumpellin occurs through at least one additional WASH complex component, KIAA1033, the demonstration of this interaction strongly suggests that the core WASH complex assembles in the brain.

We next examined whether the WASH complex interacts with retromer in the brain. VPS26 and Wash1 were immunoprecipitated in lysate prepared from rat brain tissue, using antibodies against the endogenous proteins (Fig. 2B). We found that each protein co-immunoprecipitated the other. We concluded that brain tissue has a WASH complex that interacts with retromer.

### 3.2 WASH and retromer are present in somato-dendritic and axonal compartments in primary cortical neurons

**Fig. 3:**
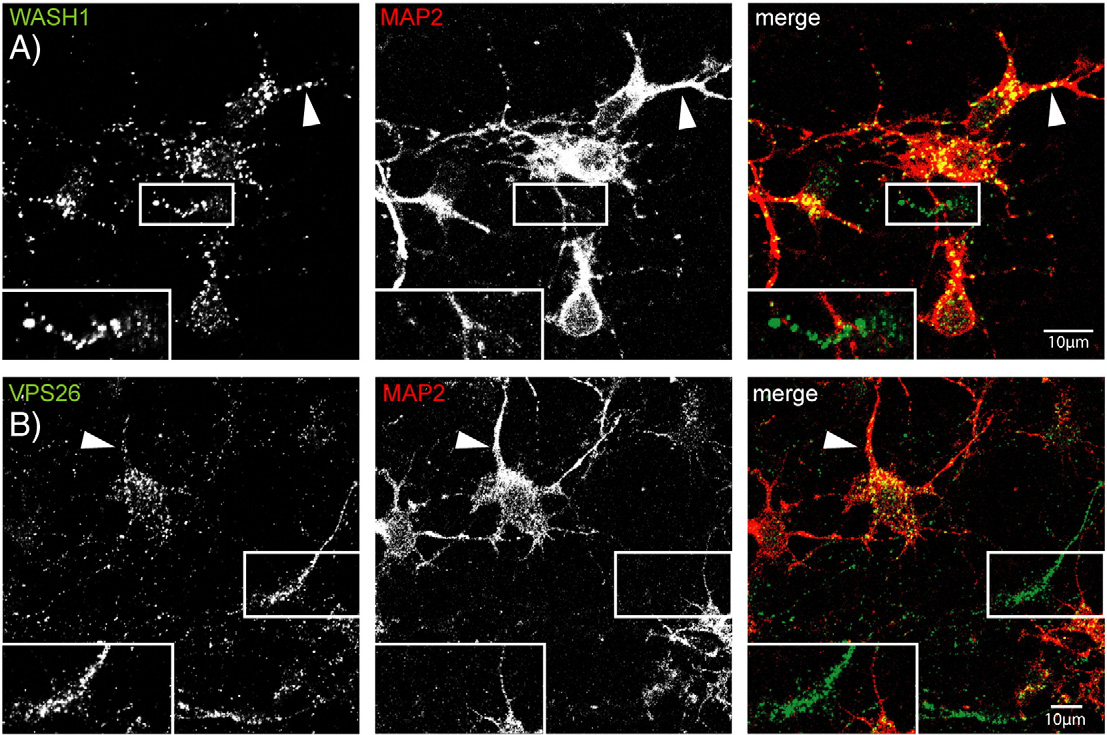
Wash1 and VPS26 localise to the dendrites and axons in neuronal cells. Rat primary cortical neurons were co-labelled with antibodies against MAP2, a marker of the cell body and dendrites and Wash1 (A) or VPS26 (B). Wash1 and VPS26 present in the MAP2 compartment, in both the cell body and dendrites (examples indicated with arrowheads). They were also present in non-MAP2 labelled regions (inset magnified images in A and B), indicating that the WASH complex and retromer are in axons. In these and subsequent images, the colour of the lettering in each greyscale panel indicates the colour of that image in the corresponding merged colour panel.

Having established the existence of the WASH complex in the brain, and that it interacts with retromer there, we examined the subcellular localisation of these two complexes in rat primary cortical neurons. We used cortical neuronal cultures as they contain upper motor neurons, the cell type affected in HSP. To establish the neuronal compartments in which the WASH and retromer complexes are present we used the MAP2 marker, which labels the cell body and dendrites but not the axons. We found that both endogenous Wash1 (Fig. 3A) and VPS26 (Fig. 3B) labelling was widespread in the cell body and extended into the dendrites. Wash1 and VPS26 puncta were also visible in non-MAP2-labelled processes, indicating that these proteins are present in axons (magnified images in Fig. 3A and B).

### 3.3 Wash1, VPS26 and strumpellin localise to EEA1-positive endosomal compartments in neurons

We investigated whether the neuronal WASH and retromer complexes localise to endosomal compartments, as shown previously in non-neuronal cell types, by labelling rat primary cortical neurons with EEA1 (early endosome antigen 1), a marker of early sorting endosomes, and either endogenous Wash1, endogenous VPS26, or myc-tagged strumpellin (strumpellin-myc). Tagged strumpellin was used as the antibody to endogenous strumpellin did not work for immunofluorescence.

**Supplementary Fig. 1:**
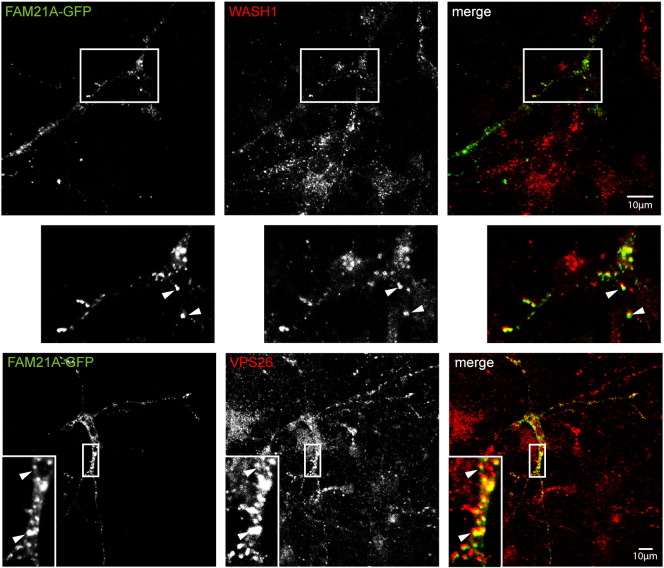
WASH complex member FAM21 co-localises with Wash1 and retromer in neuronal cells. GFP-tagged FAM21 was transiently transfected into rat primary cortical neurons and the cells were labelled with the markers indicated.

**Fig. 4:**
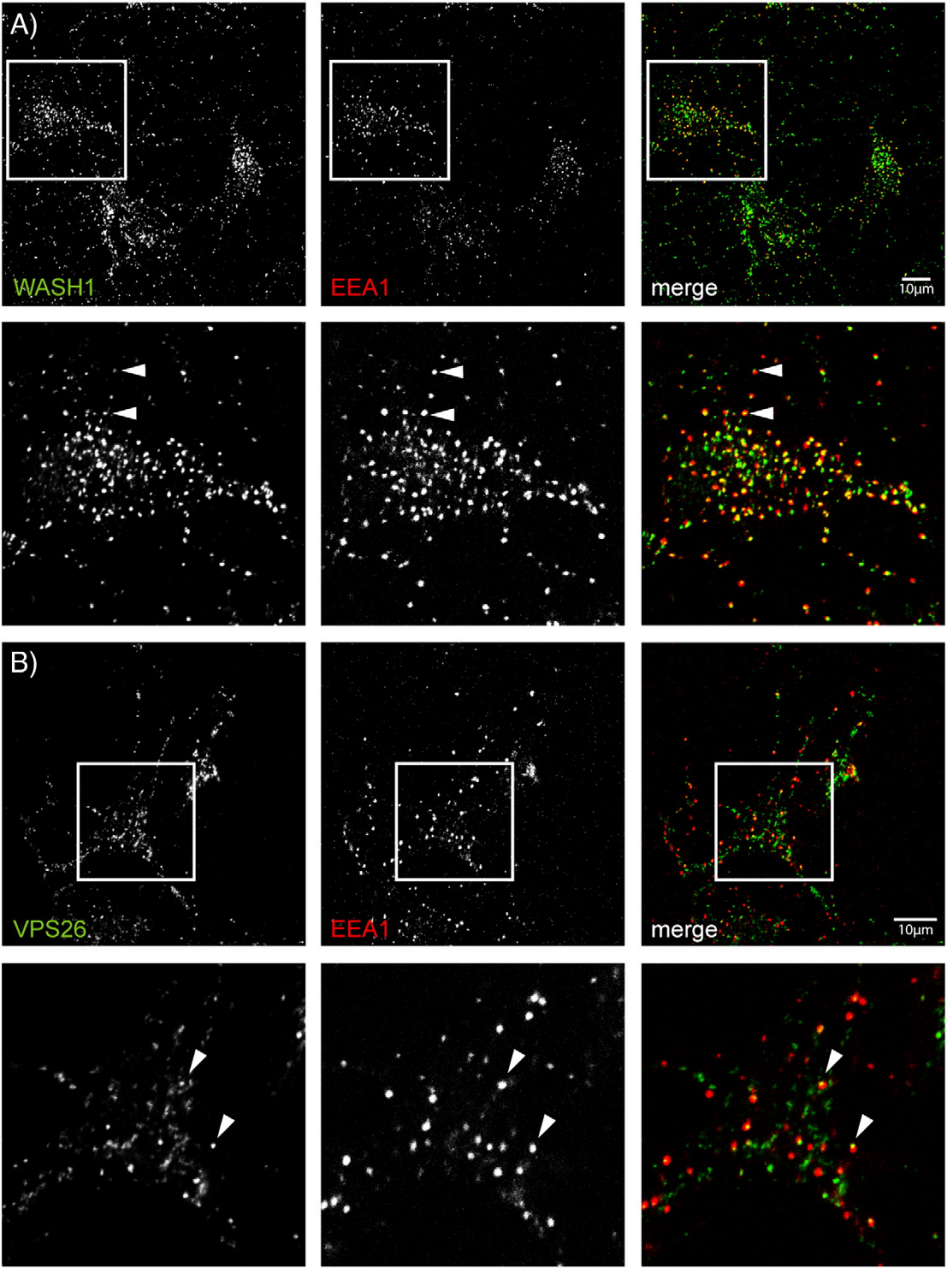

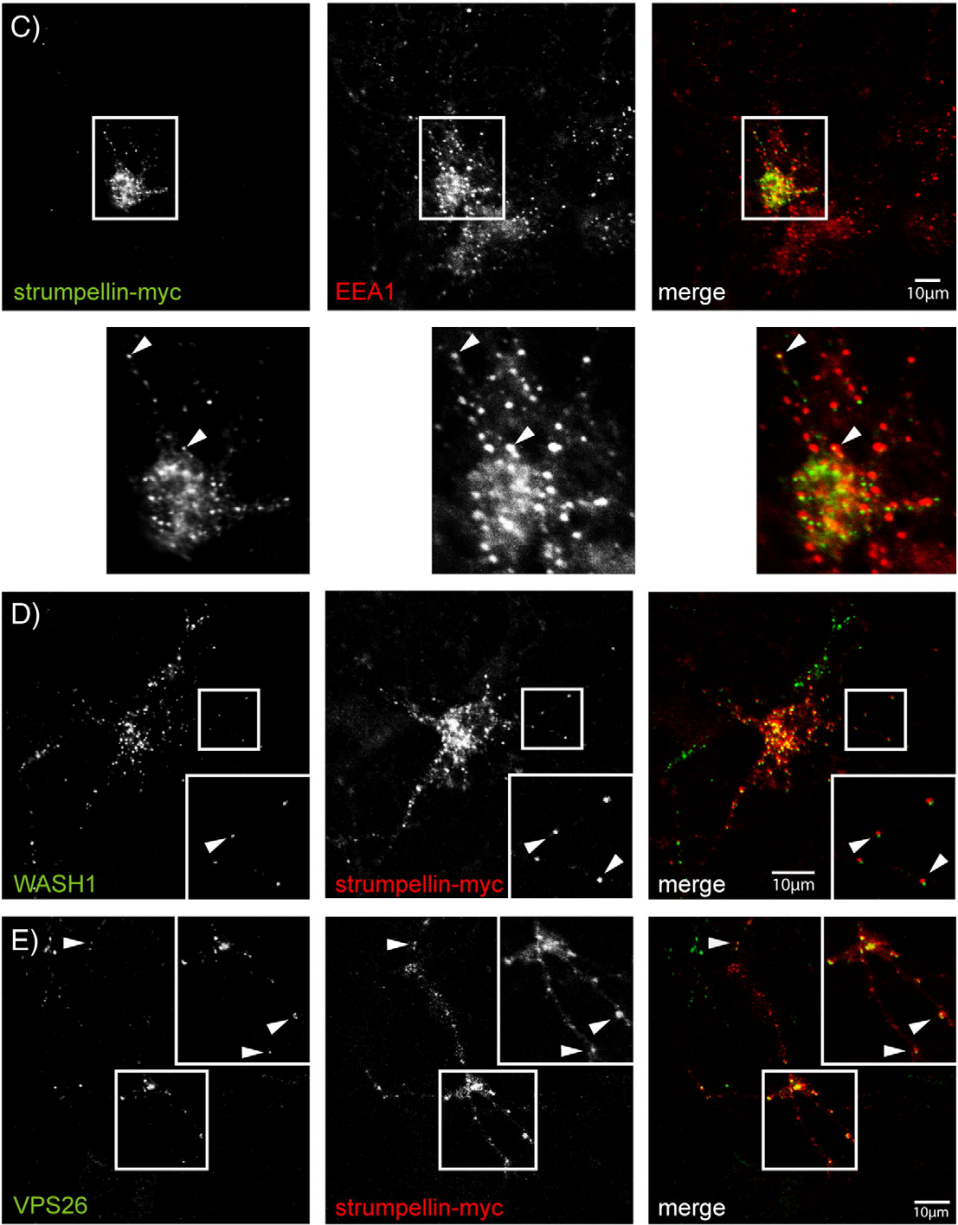
Wash1 and VPS26 localise to the early endosomes in neurons. Rat primary cortical neurons were labelled with the markers indicated. Examples of puncta showing co-localisation are indicated by arrowheads. In the case of cells labelled with EEA1 and Wash1 (A) or EEA1 and VPS26 (B), the arrowheads indicate puncta in which the WASH or retromer complex marker appeared to label a subdomain of the EEA1 compartment. The magnified images (inset or below the labelled panels) correspond to the boxes indicated on the labelled figure panels.

Wash1 co-localised extensively with EEA1. It was often visible as peripheral spots on EEA1-positive puncta, consistent with reports in several non-neuronal cell types showing that Wash1 localises to restricted domains of the sorting endosomes (Fig. 4A) [9,10]. VPS26 displayed a similar restricted location on domains of sorting endosomes (Fig. 4B). Strumpellin-myc also showed co-localisation with EEA1 (Fig. 4C). In addition, we saw extensive co-localisation between pairs of WASH complex members. Thus, for example, strumpellin-myc strongly co-localised with endogenous Wash1 and VPS26 (Fig. 4D, E), and FAM21 colocalised with Wash1 (Supplementary Fig. 1). We concluded from these studies that the WASH complex is present on retromer-positive early endosomes in neurons.

### 3.4 Disease mutation of strumpellin does not prevent its participation in the WASH complex or interaction with retromer

**Fig. 5:**
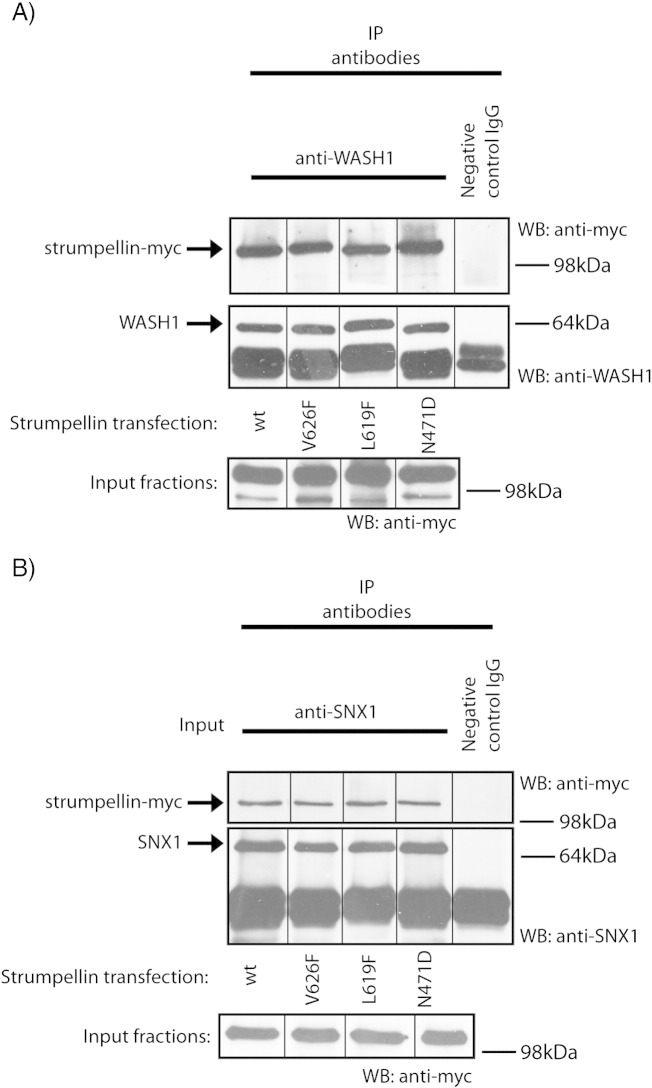
Both wild type and disease mutant strumpellin co-immunoprecipitate with WASH and retromer complex components. Lysates prepared from HeLa cells transfected with either wild type or disease mutant strumpellin were used in co-immunoprecipitation experiments with either anti-Wash1 (A) or anti-SNX1 (B) as the IP antibody. Spurious lanes on the blots have been spliced out, as indicated by the vertical lines.

Having shown that the interaction and localisation of WASH complex members are comparable in neurons and non-neuronal cells, we next examined the effect of strumpellin mutation on its interactions, localisation and known cellular functions. We began by examining whether strumpellin mutation affected its participation in the WASH complex. When Wash1 was immunoprecipitated in HeLa cells transfected with myc-tagged wild-type or disease mutant strumpellin, all forms of strumpellin were co-immunoprecipitated in equal amounts (Fig. 5a).

We next examined whether disease mutant forms of strumpellin still allowed the interaction between the WASH complex and the retromer complex. We immunoprecipitated endogenous SNX1 in HeLa cells expressing wild-type or mutant forms of strumpellin. All forms of strumpellin associated with SNX1 in apparently equal amounts (Fig. 5B).

**Supplementary Fig. 2:**
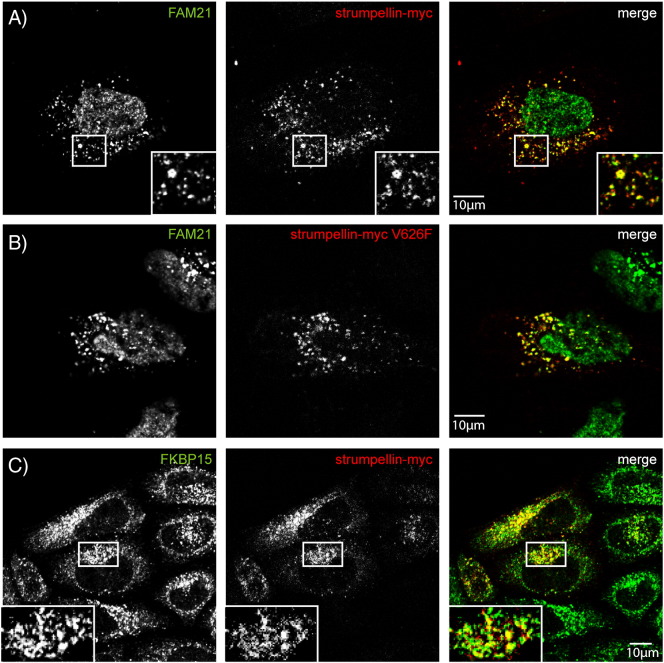

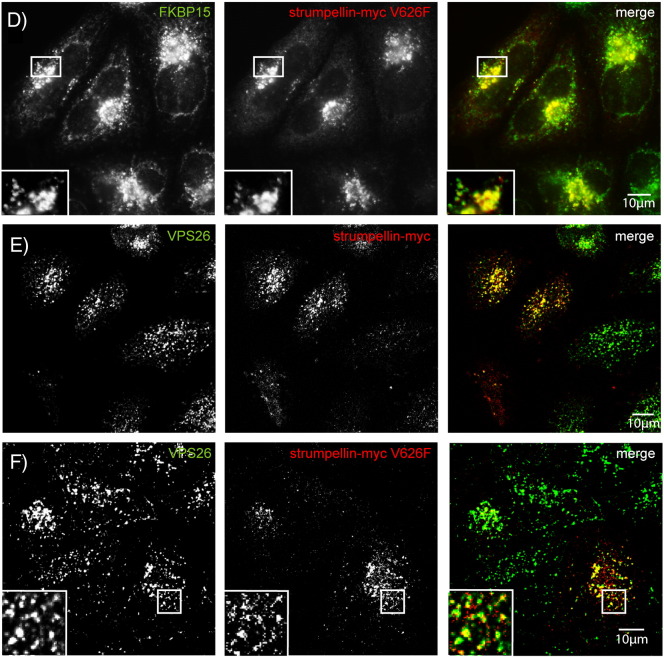
Wild-type and disease mutant strumpellin co-localise with WASH and retromer markers in HeLa cells. HeLa cells were transiently transfected with myc-tagged wild-type strumpellin (A, C, E) or disease mutant strumpellin-myc V626F (B, D, F) and labelled with the markers indicated.

**Supplementary Fig. 3:**
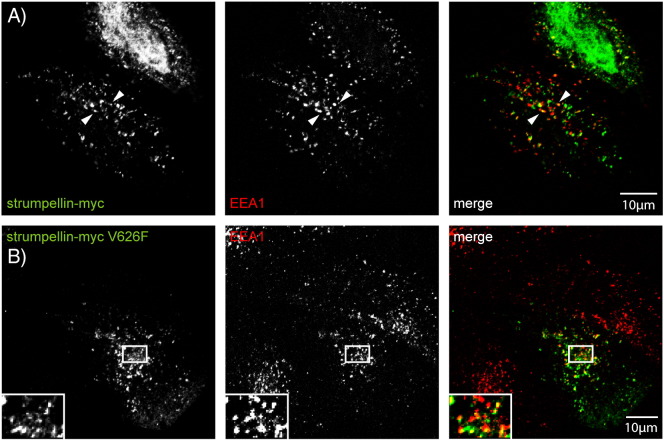
Wild-type and disease mutant strumpellin co-localise with EEA1 in HeLa cells. HeLa cells were transiently transfected with myc-tagged wild-type strumpellin (A) or disease mutant strumpellin-myc V626F (B) and labelled with the early endosomal marker EEA1.

**Fig. 6:**
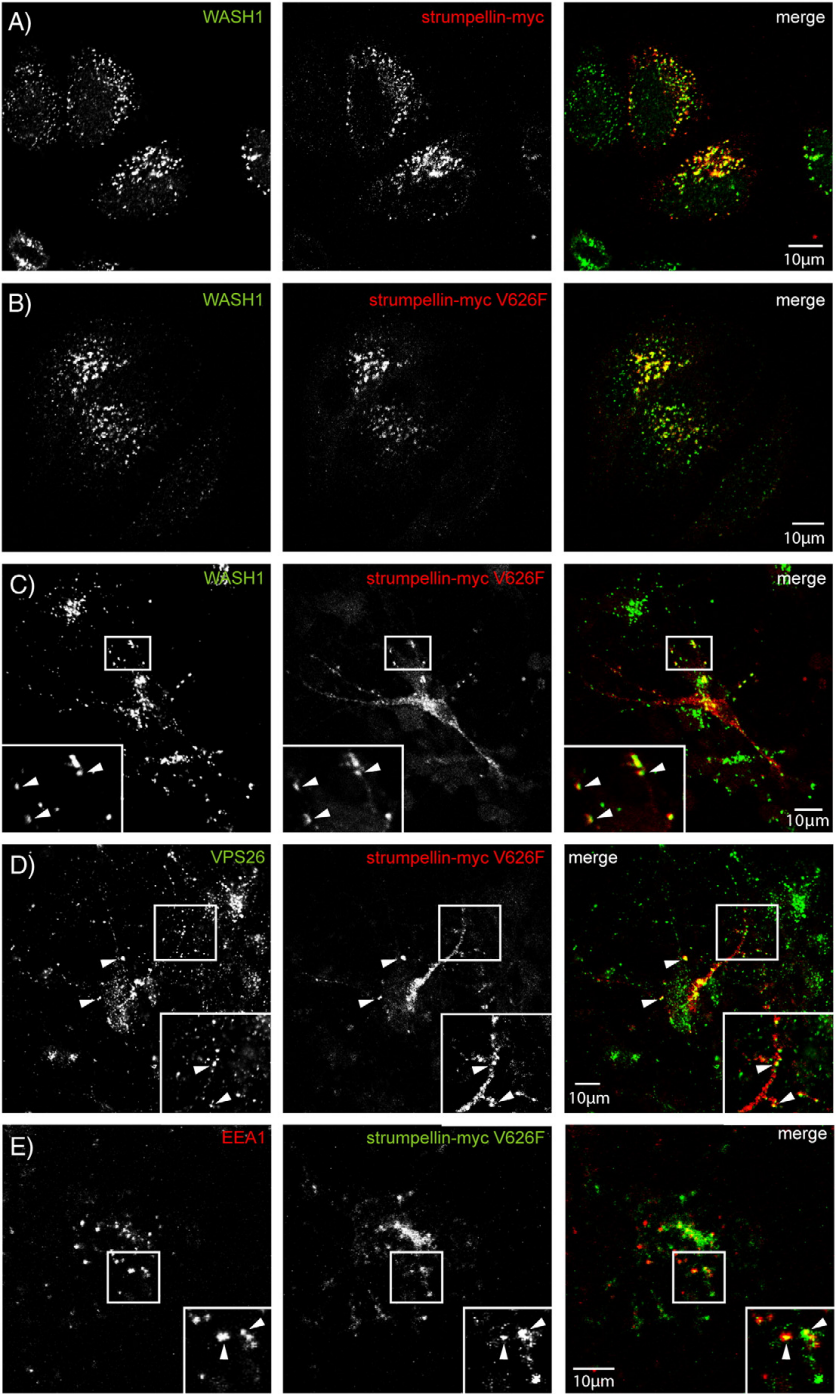
Wild-type and disease mutant strumpellin co-localises with WASH, retromer and EEA1 in neurons. HeLa cells (A and B) or rat primary cortical neurons (C–E) were transiently transfected with myc-tagged wild-type strumpellin (A) or disease mutant strumpellin-myc V626F (C–E), and labelled with the markers indicated. The arrowheads indicate examples of co-localised puncta.

Consistent with these results, strumpellin mutation did not affect the co-localisation of the protein with other WASH or retromer complex components, as both wild-type and mutant strumpellin showed similar co-localisation with Wash1 (Fig. 6A, B), VPS26, FAM21 and FKBP15 (Supplementary Fig. 2) in HeLa cells and with Wash1 and VPS26 in primary cortical neurons (Fig. 6C, D). Data are presented for the V626F mutation, although essentially similar results were found for the other two mutations. In addition, mutation of strumpellin did not affect its location on endosomes, as wild-type and mutant strumpellin showed similar patterns of co-localisation with EEA1, in primary cortical neurons (Figs. 4C and 6E) and in HeLa cells (Supplementary Fig. 3). Knockdown of strumpellin causes mislocalisation and perinuclear clustering of other members of the WASH complex, notably WASH [13]. However, this did not occur after transfection of mutant strumpellin (Fig. 6B).

We concluded that mutation of strumpellin does not affect its incorporation into the WASH complex, the ability of the WASH complex to interact with retromer, or the subcellular distribution of the complex.

### 3.5 Expression of strumpellin mutants does not cause endosomal tubulation

**Fig. 7:**
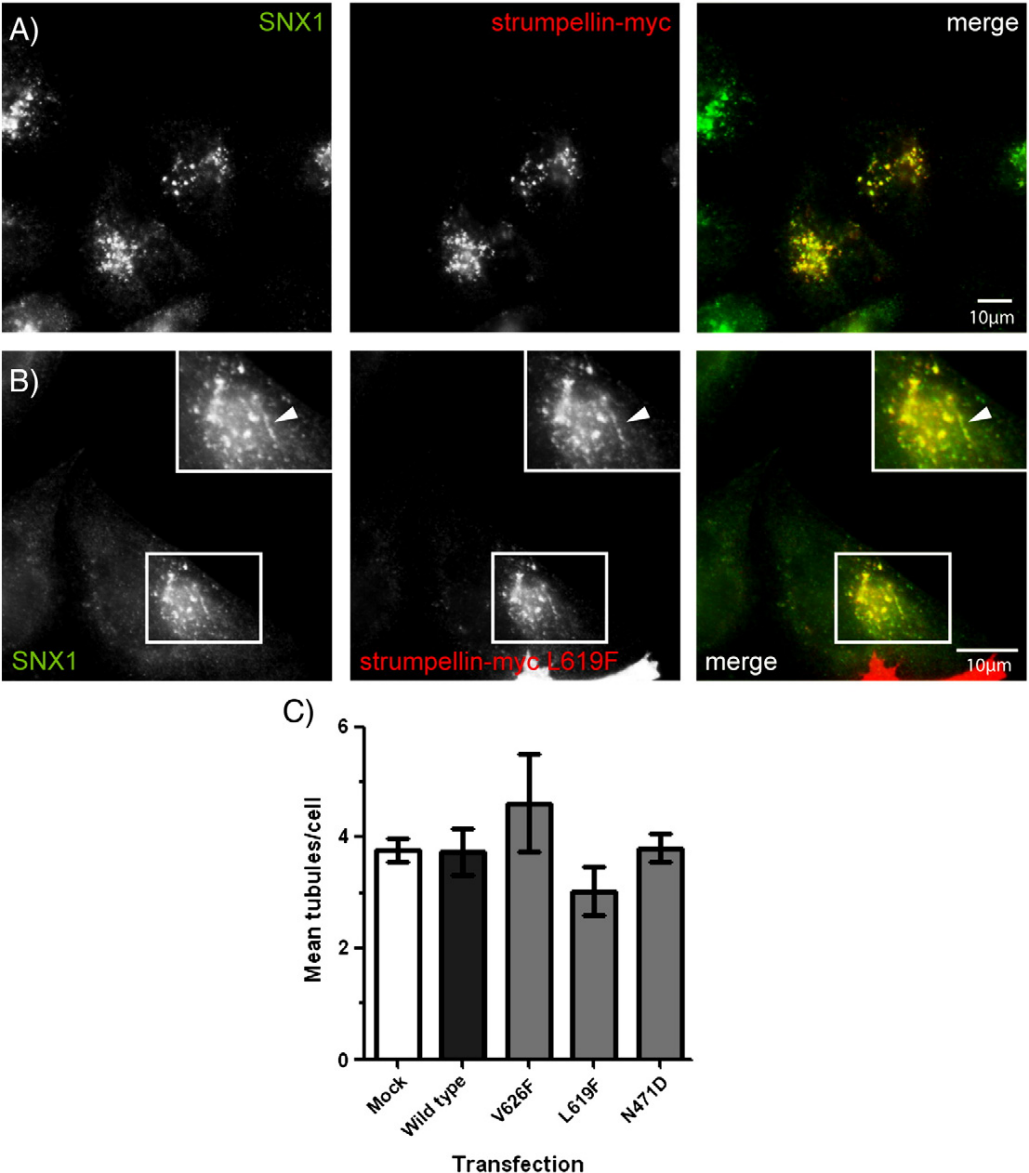
Strumpellin mutation does not induce endosomal tubulation. HeLa cells were transfected with strumpellin-myc (A) or strumpellin-myc-L619F (B), then labelled with SNX1. In (B) the arrowheads indicate a tubular structure. (C) The mean number of SNX1 tubules per cell was counted in mock transfected cells (Mock) and in cells transfected with wild type strumpellin-myc (Wild type) or with the disease mutants strumpellin-myc V626F (V626F), strumpellin-myc L619F (L619F) and strumpellin-myc N471D (N471D). Error bars represent S.E.M., n = 3 experiments.

Mammalian cells lacking strumpellin have an increased number of SNX1- and VPS26-positive endosomal tubules [9,10,13]. We examined whether over-expression of mutant strumpellin might cause a similar effect. Both wild-type and mutant strumpellin-myc showed strong co-localisation with SNX1 (Fig. 7A, B), and in some cases this co-localisation was on SNX1-positive tubules (arrowheads in Fig. 7B). However, quantitation of the number of SNX1 tubules revealed no significant difference in cells expressing wild-type strumpellin versus mutant forms of the protein (Fig. 7C).

### 3.6 Strumpellin depletion, but not expression of strumpellin mutants, alters the localisation of SNX27 and β2AR

The WASH complex functions with SNX27 to package β2AR into retromer-positive endosomal tubules for recycling to the plasma membrane [20]. We examined the effect of strumpellin depletion and mutation on these proteins, as it has not been characterised previously.

**Supplementary Fig. 4:**
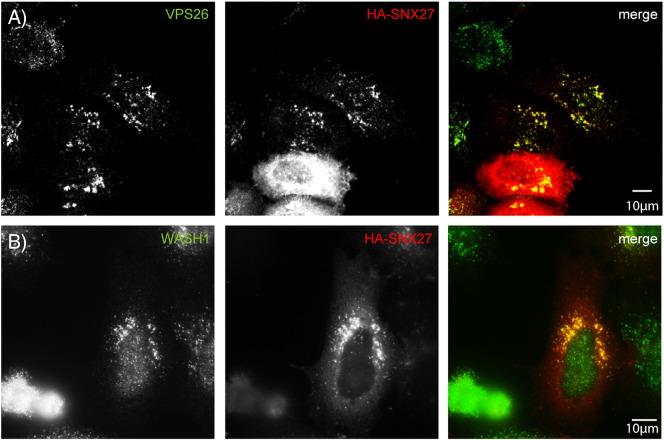
SNX27 co-localises with retromer markers. HeLa cells were transfected with HA-tagged SNX27 and labelled with VPS26 (A) or Wash1 (B).

**Supplementary Fig. 5:**
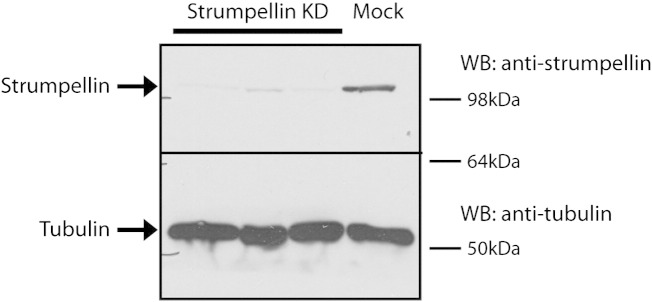
Strumpellin siRNA transfection depletes strumpellin from HeLa cells. Cell lysates prepared from cells transfected with strumpellin siRNA (n = 3 experiments) or from mock-transfected cells were immunoblotted with anti-strumpellin. Blotting versus tubulin serves as a loading control. This experiment also serves as validation that the signal detected by the anti-strumpellin antibody is specific. This strumpellin band runs at approximately 120 kD.

**Fig. 8:**
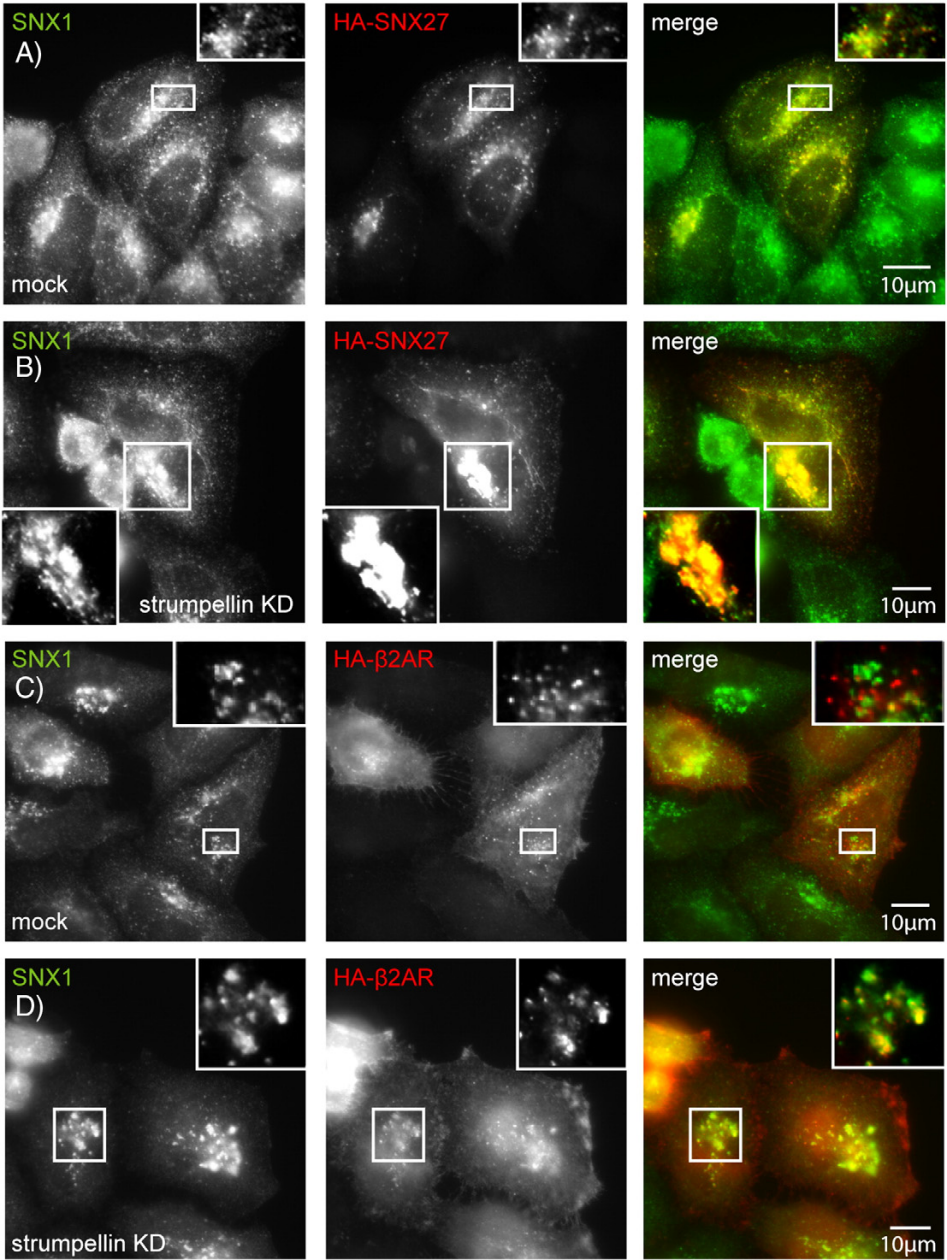

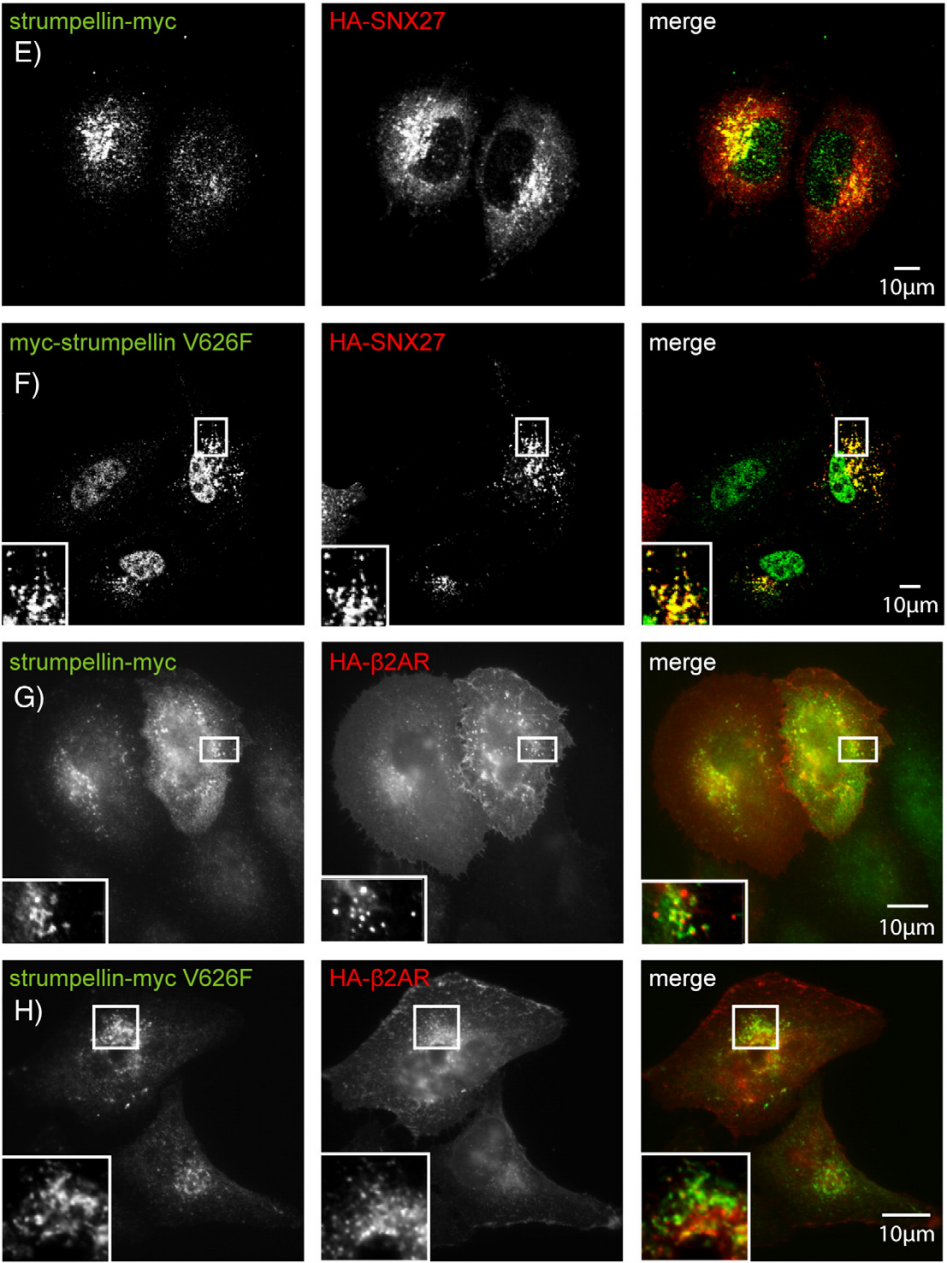
Strumpellin depletion, but not mutation, alters the distribution of SNX27 and β2AR. (A–D) HeLa cells were subjected to mock siRNA transfection (A and C) or were transfected with siRNA versus strumpellin (B and D), and in addition were transfected with HA-SNX27 (A and B) or HA-β2AR (C and D). (E–H) HeLa cells were transfected with wild-type strumpellin-myc (E and G) or with strumpellin-myc V626F (F and H), as well as HA-SNX27 (E and F) or HA-β2AR (G and H). Strumpellin knock-down was confirmed by immunoblotting (Supplementary Fig. 5).

We began by confirming that in our hands HA-tagged SNX27 co-localised in puncta with WASH and retromer complex markers (Supplementary Fig. 4). We then examined the distribution of HA-SNX27 in cells lacking strumpellin and found that SNX27-positive structures appeared larger and showed more peri-nuclear clustering than in control cells (Fig. 8A, B, Supplementary Fig. 5). The clustered SNX27 puncta strongly co-localised with SNX1 (Fig. 8B). Consistent with the known role of SNX27 in traffic of β2AR, we also found that in cells lacking strumpellin the distribution of this receptor was altered in a similar way to that seen with SNX27. In control cells HA-tagged β2AR was visible as cytoplasmic puncta that showed minor colocalisation with SNX1 (Fig. 8C). However, in cells lacking strumpellin the β2AR puncta appeared enlarged, clustered near the nucleus, and showed strong co-localisation with SNX1 (Fig. 8C, D).

We then examined the effect of mutant strumpellin on SNX27 and β2AR distribution. Although wild-type and mutant strumpellin co-localised with both proteins, expression of disease mutant strumpellin did not cause the altered subcellular distribution that we observed after strumpellin depletion (Fig. 8E–H).

### 3.7 Depletion or mutation of strumpellin does not affect transferrin uptake or recycling

**Fig. 9:**
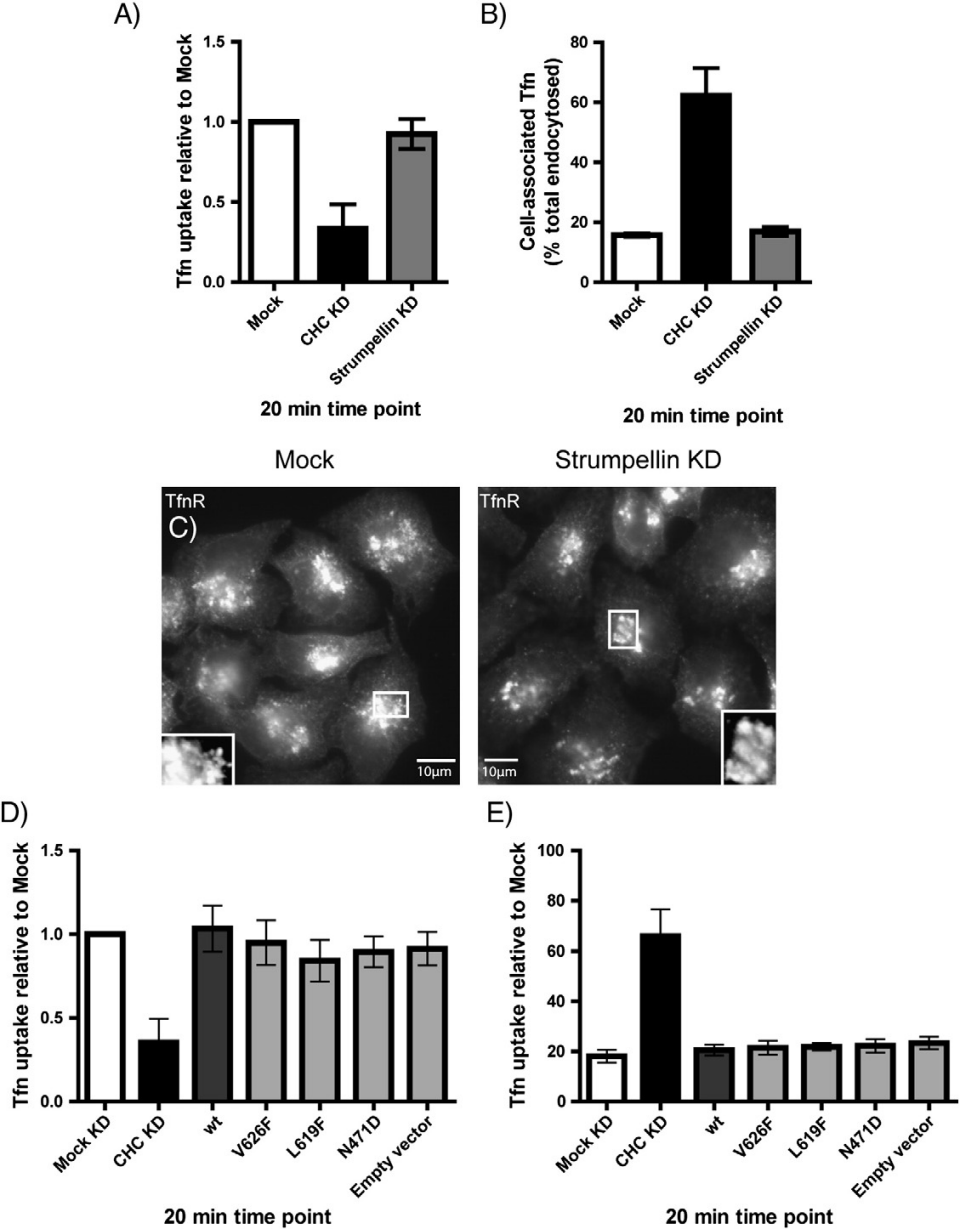
Strumpellin depletion or mutation does not affect the uptake or recycling of the transferrin receptor. HeLa cells depleted of either strumpellin or clathrin heavy chain (as a positive control) were incubated with fluorescent transferrin and used in either uptake (A) or recycling (B) assays. The amount of internalised transferrin present after 20 min in each assay was measured by flow cytometry. To examine the steady-state distribution of the transferrin receptor, mock-transfected cells or cells transfected with siRNA against strumpellin were labelled for TfnR (C). Cells transfected with wild-type or disease mutant strumpellin, or with an empty vector, were also used in uptake (D) and recycling (E) assays, with depletion of the clathrin heavy chain used as a positive control. Error bars = S.E.M, n = 3 experiments in (A), (B), (D) and (E).

Depletion of Wash1 causes defective transferrin recycling in HeLa cells, where in a FACS-based assay internalised fluorescent transferrin was less efficiently released from HeLa cells lacking Wash1 [9]. We therefore examined whether depletion of strumpellin affected transferrin endocytosis or recycling, using a well-established FACS-based assay. However, we found no effect of strumpellin depletion on transferrin uptake or recycling in HeLa cells (Fig. 9A, B). In addition, the steady state subcellular distribution of the TfnR was not altered in HeLa cells lacking strumpellin (Fig. 9C). We also examined the effect of disease mutation of strumpellin on transferrin uptake and recycling, and found no obvious difference between wild-type and mutant strumpellin (Fig. 9D, E).

## 4 Discussion

### 4.1 Strumpellin and the function of the WASH complex in the brain

In view of the involvement of strumpellin in the axonopathy associated with HSP, it is critical to understand the role of this protein in neurons. We have shown biochemically that the WASH complex assembles in brain tissue. This supports the findings of Jia et al., but we have extended these earlier studies with novel data to show that in the brain the WASH complex interacts with retromer, and that the WASH and retromer complexes are present on punctate structures in both the somatodendritic and axonal compartments [15]. Furthermore, we show that the WASH complex and retromer are present on early endosomes in neurons. Thus, the localisation and basic interactions of the WASH complex in neurons appear very similar to that described in other cell types. We therefore consider it highly likely that the neuronal WASH complex will participate in the control of actin networks, to regulate endosomal tubule fission events that depend on the actin cytoskeleton. As both the WASH complex and retromer were present in the somato-dendritic and axonal compartments, our work indicates that such processes are important throughout the neuron, including at the site of HSP pathology in the axon.

A functional consequence of defective endosomal tubule fission is altered traffic of receptors that normally transit endosomal tubular compartments, and this could cause HSP if receptors important for axonal survival are affected. We examined the role of strumpellin in the traffic of selected receptors, to identify new functions of the protein. Wash1 depletion has a slight inhibitory effect upon recycling of TfnR [27,28], but we found no effect of strumpellin depletion on this pathway. The WASH complex is also implicated in β2AR recycling as it interacts with SNX27, an adapter that links β2AR to retromer-positive tubules [29]. However, a requirement for the WASH complex in β2AR recycling had not been demonstrated. We found that β2AR and its cargo adaptor SNX27 had an altered subcellular distribution and increased localisation to retromer-positive compartments in cells lacking strumpellin, consistent with a failure to package β2AR into tubules for recycling to the plasma membrane. There are neuronal cargoes that could be affected by abnormality of this SNX27 pathway, for example, SNX27 has a role in trafficking subunits of the Kir3 potassium channel in hippocampal neurons [30], and clearly receptors trafficked by this pathway are candidates to be investigated for involvement in HSP.

Bone morphogenic protein receptors (BMP) are also good candidates to be affected by strumpellin mutation. Fly larvae lacking a number of endosomal or actin-regulating proteins have neuromuscular (NMJ) synaptic overgrowth, characterised by the appearance of “satellite boutons”. This phenotype is caused by upregulated BMP signalling, leading to the proposal that endosomal processes requiring actin-based force generation are important in attenuating BMP signalling. The best-characterised protein involved in this pathway is the recycling endosomal protein nervous wreck. This protein contains a membrane-tubulating F-BAR domain, binds to dynamin, and also binds to Wasp, which in turn regulates actin assembly by activating the Arp2/3 complex. Nervous wreck binds to the BMP receptor thickveins, and via an interaction with SNX16, attenuates signalling by activated BMP receptors [31,32]. The role of endosomal tubulating domains and actin dynamics in this pathway raises the intriguing possibility that strumpellin and the WASH complex could also be involved in regulating BMP receptor traffic and signalling at the NMJ. This idea is strengthened by the observation that flies lacking Vps35, the retromer component that interacts with the WASH complex, have NMJ synaptic overgrowth and satellite boutons. This phenotype was caused by upregulated BMP signalling and accompanied by altered actin regulation [33]. Against this background it is significant that a number of HSP proteins are inhibitors of BMP signalling in model organisms or in cultured cells, and upregulated BMP signalling has been proposed as a unifying pathogenic mechanism for HSP [34–36]. It will be important in the future to examine whether the WASH complex is involved in this process, and in particular whether strumpellin mutations affect BMP trafficking and signalling in neuronal systems.

### 4.2 The pathological mechanism of strumpellin mutation

We explored a number of possible mechanisms by which strumpellin mutations could exert a pathological effect. We first examined whether strumpellin mutation might prevent incorporation of strumpellin into the WASH complex. However, since mutant and wild-type forms of strumpellin co-immunoprecipitated with Wash1 with equal efficiency, this is not likely. These results support those of Jia et al., who also found that mutant strumpellin could support WASH complex formation [15]. It remains possible that mutation of strumpellin might specifically affect recruitment of an as yet unidentified accessory, “non-core” protein to the complex, and this requires to be systematically examined as new proteins associated with the WASH complex are identified.

Although mutant strumpellin did not destabilise the WASH complex, it remained possible that the mutant protein could alter the complex so that it was unable to bind retromer or localise to endosomes. However, our experiments demonstrated that mutant strumpellin could be co-immunoprecipitated with a retromer component. Consistent with this, disease mutant strumpellin was still able to localise to endosomes and to co-localise with other WASH and retromer complex components, both in neuronal cells and in HeLa cells. Thus mutant strumpellin is able to participate in the WASH complex, and does not alter the localisation of the WASH complex or retromer on endosomes.

HeLa cells lacking strumpellin have increased tubulation of the SNX1 and retromer endosomal compartment [10,13]. Cells depleted of other WASH complex components, such as Wash1, also have increased endosomal tubulation, and this phenotype has been interpreted as a consequence of the loss of a WASH complex-generated pushing force involved in endosomal tubule fission [9,10]. Since mutant strumpellin is incorporated into the WASH complex, we asked whether this might cause endosomal tubulation by exerting a dominant negative effect on the complex. However, we found no increase in endosomal tubulation in cells expressing mutant strumpellin, even though the mutant protein was localised appropriately on endosomes and endosomal tubules. These results are consistent with findings in zebrafish, where expression of disease-mutant strumpellin in animals in which endogenous strumpellin was present did not cause a phenotype [8].

What then is the likely pathological mechanism of strumpellin mutation? The mutational spectrum seen in SPG8 HSP is very specific, consisting of only a limited number of missense mutations and no nonsense mutations, frameshift mutations or whole exon deletions [8]. This argues against a haplo-insufficiency pathological mechanism, with the caveat that additional mutational classes may be found as more HSP families are screened for strumpellin mutations. Models of the stoichiometry of the WASH complex suggest that there is 1 strumpellin molecule in the complex [9,15]. Since mutant strumpellin is expressed and incorporated into the WASH complex, cells transiently expressing mutant strumpellin will have a mixed population of WASH complexes, some containing endogenous wild-type strumpellin and others mutant strumpellin. While incorporation of mutant strumpellin might have a deleterious effect on the individual complex involved, it is possible that sufficient “normal” WASH complexes remain to allow proper endosomal tubule fission. Consistent with the idea that complexes containing mutant strumpellin are non-functional, human wild-type, but not mutant, strumpellin could rescue the curly tail phenotype seen in zebrafish lacking strumpellin [8]. In the human disease, complexes containing wild-type and mutant strumpellin should be present in equal numbers. We propose that in most cell types, sufficient functional WASH complexes would be present to allow normal cellular physiology. However, owing to the special demands on membrane traffic imposed by their exceptionally long axons, we suggest that corticospinal neurons are especially vulnerable to reductions in functional WASH. It is at first sight perplexing why haplo-insufficiency mutations would not also cause the disease if WASH complexes containing mutant WASH are non-functional. We speculate that haplo-insufficiency mutations might lead to a compensatory up-regulation of strumpellin expression from the *trans* normal allele or a decrease in protein degradation, which might not be triggered in the presence of a normal overall number of WASH complexes. Experimental approaches are now available to test these hypotheses.

In summary, in this study we show that the WASH complex assembles in the brain, and that in primary neurons it is present in somatodendritic and axonal compartments and localises to endosomes. Furthermore, we show that disease causing mutations do not affect the assembly of the WASH complex. Mutations do not have a dominant effect on the known functions of strumpellin in endosomal tubulation, although we speculate that strumpellin mutations have a deleterious effect on individual complexes. These observations provide a platform for further studies aimed at conclusively determining the pathological mechanism of strumpellin mutation, and understanding the normal and pathological roles of the protein in the brain.

The following are the supplementary data related to this article.

## Figures and Tables

**Table 1:** Proteins reported as interacting with the WASH complex or strumpellin.

WASH complex/strumpellin/retromer interacting protein	Publication
AP2	[37]
Capping protein (CP) Zα and β	[9]
Coiled-coil domain containing 53 (CCDC53)	[9]
Dynamin 2	[9]
Tubulin	[10]
FKBP15	[13]
TBC1D5	[13]
Valosin-containing protein (VCP)	[14]
SNX27	[20]
CCDC22	[19]
CCDC93	[19]
